# Clinical Features of Glutamic Acid Decarboxylase‐65 Neurological Autoimmunity: A Case Series From China

**DOI:** 10.1111/cns.70237

**Published:** 2025-02-20

**Authors:** Zhandong Qiu, Fang Xu, Mengyao Zhang, Xixi Yang, Yan Han, Dawei Li, Liang Liu, Jia Chen, Lehong Gao, Qing Xue, Yue Hou, Ying Sun, Li Di, Chunqiu Fan, Junhua Liang, Yue Han, Huiqing Dong, Junwei Hao, Zheng Liu

**Affiliations:** ^1^ Department of Neurology Xuanwu Hospital Capital Medical University, National Center for Neurological Disorders Beijing China

**Keywords:** epilepsy, glutamic acid decarboxylase‐65, immunotherapy, limbic encephalitis, rituximab

## Abstract

**Objective:**

To explore the clinical phenotypes, characteristics, immunotherapy response, and outcomes of glutamic acid decarboxylase‐65 (GAD65) neurological autoimmunity.

**Methods:**

We performed a retrospective review of patients diagnosed with GAD65 neurological autoimmunity in the Department of Neurology at Xuanwu Hospital over the past 6 years (2017–2023). The clinical and laboratory data, imaging, therapeutic response, and long‐term prognosis of those patients were collected and analyzed.

**Results:**

Among the 37 patients displaying significant neurological impairment, there were 14 males (37.8%) and 23 females (62.2%), with a median age of onset of 41.5 (25–59) years and a median interval of 9 (1.5–36) months from onset to a definitive diagnosis. Clinical phenotypes included epilepsy (15, 40.5%), limbic encephalitis (7, 18.9%), brainstem dysfunction (2, 5.4%), parkinsonism (2, 5.4%), peripheral neuropathy (3, 8.1%), cerebellar ataxia (1, 2.7%), and overlap syndromes (7, 18.9%). Out of 36 patients who received immunotherapy, the median time from onset to initiation of immunotherapy was 8.5 (1.5–37.5) months. Four cases were lost to follow‐up, leaving a median follow‐up period of 20.5 (16–37.25) months among the remaining 32 patients. Most patients (26, 81.3%) responded positively to immunotherapy, with some showing mild improvement or no response. Some patients showed inadequate responses to treatments such as mycophenolate mofetil (MMF), but significant improvement is observed after switching to rituximab (RTX). The relationship between the timing of initiating immunotherapy and prognosis by Spearman's rank correlation only showed weak correlation.

**Conclusion:**

The clinical spectrum of GAD65 neurological autoimmunity appeared highly diverse. Immunotherapy can benefit the majority of patients, and early treatment appeared to be associated with good prognosis. RTX may be more effective than MMF; however, this requires more rigorous prospective studies to explore.

## Graphical abstract

The clinical spectrum of GAD65 autoimmune neurological diseases appeared highly diverse. Immunotherapy can benefit the majority of patients, and early treatment appeared to be associated with good prognosis. RTX may be more effective than MMF; however, this requires more rigorous prospective studies to explore.

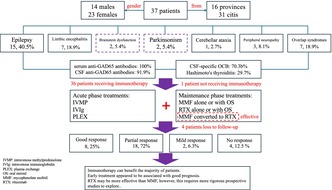

## 1 Introduction

GAD65 is an intracellular enzyme crucial for converting glutamate to gamma‐aminobutyric acid (GABA), a major inhibitory neurotransmitter in the central nervous system (CNS) [1, 2]. Antibodies against GAD65 are frequently detected in various neurological disorders and in type 1 diabetes mellitus (T1DM) [3]. Current reported phenotypes include epilepsy, limbic encephalitis, cerebellar ataxia, and overlap syndromes [2, 4, 5]. Brainstem dysfunction, spinal cord and cognitive impairment are considered as secondary manifestations of GAD65 neurological autoimmunity, often co‐occurring with classical phenotypes, although isolated brainstem dysfunction cases are not well‐documented [5]. Phenotypes like parkinsonism and peripheral neuropathy have been sporadically reported [6, 7], necessitating more cases to firmly establish their association with GAD65 antibodies.

The pathophysiologic role of anti‐GAD65 in neuroinflammation is still controversial [8, 9, 10]. It is hard to understand whether there is a direct antibody‐associated pathogenic effect because the target antigen is located intracellularly. Moreover, responses to immunotherapy seem to be poorer than in patients with neurologic disorders caused by most other antineuronal antibodies [11, 12]. More studies are needed to evaluate the effectiveness and prognosis of current treatment regimens.

Historically, methods for detecting GAD65 antibodies have varied, with a significant portion of studies relying on ELISA or radioimmunoassay. In this study, all patients underwent cell‐based assay (CBA) testing, complemented by ELISA and immunoblotting in some cases. Throughout follow‐up, serum and cerebrospinal fluid (CSF) antibodies were reassessed in some patients to monitor changes in titers of GAD65 antibodies under immunotherapy. Given the aforementioned background, we focused on evaluating the demographic characteristics, clinical presentations, immunotherapy response, and long‐term prognosis of GAD65 neurological autoimmunity diagnosed at Xuanwu Hospital in recent years, aiming to enhance disease spectrum comprehension and offer new insights for clinical diagnosis and treatment.

## 2 Methods

### 2.1 Patients

We have collected detailed information on 37 patients diagnosed with GAD65 neurological autoimmunity, admitted and treated at the Neurology Department of Xuanwu Hospital, Capital Medical University, from June of 2017 to December of 2023; these patients were subsequently followed up. All patients exhibited clinical manifestations indicative of nervous system involvement, tested positive for anti‐GAD65 antibodies in blood and/or CSF, and underwent thorough evaluations to exclude other potential etiologies by neurologists. Informed written consent was acquired from patients or their representatives.

We provide comprehensive descriptions of the patients' demographic characteristics, clinical phenotypes, laboratory findings, imaging results, time from symptom onset to initial immunotherapy, and disease outcomes. Disease severity at the nadir after onset and neurological functional status at the last follow‐up were assessed using the modified Rankin Scale (mRS), Mini‐Mental State Examination (MMSE), and Montreal Cognitive Assessment (MoCA) scales depending on different phenotypes.

### 2.2 Outcome Measures

Response to immunotherapy (steroids, intravenous immunoglobulin (IVIg), plasma exchange (PLEX), mycophenolate mofetil (MMF), and rituximab) was classified as no response, mild response, partial response (i.e., minimal residual clinical signs/symptoms), or good response (i.e., no residual clinical signs/symptoms). The assessment of treatment response was carried out by two clinicians, F Xu and My Zhang. If there is a disagreement, it will be further evaluated by Zd. Qiu until a consensus is reached.

### 2.3 Laboratory Investigations

Upon admission, all patients underwent lumbar puncture, with CSF analyzed for white cell count, protein levels, and specific oligoclonal bands (OCB). Routine diagnostic workup for suspected neuroimmune disorders and encephalitis included assessing antibodies associated with autoimmune encephalitis, paraneoplastic neurological syndromes, and CNS demyelinating diseases via CBA in both serum and CSF. Additionally, some patients were concurrently tested using ELISA and immunoblotting methods. Serum antinuclear antibodies (ANA), antineutrophil cytoplasmic antibodies, anticardiolipin antibodies, and rheumatic factor (RA) were tested in all patients to exclude systemic autoimmunity and to seek concomitant autoantibodies. All patients were screened for diabetes and thyroid disease. CSF samples from all patients were also screened for viruses and bacteria to exclude infections in the CNS.

### 2.4 Imaging

All patients underwent cerebral MRI examinations, encompassing T1‐weighted imaging, T2‐weighted imaging, FLAIR sequences, diffusion‐weighted imaging, and gadolinium‐enhanced T1‐weighted imaging. Positron emission tomography/computed tomography (PET/CT) scans were conducted for 12 patients. Our analysis focused on lesion localization, signal intensity, metabolic profiles, and relevant imaging parameters.

### 2.5 Statistical Analysis

This study constituted a follow‐up investigation. For variables that disobeyed Gaussian distribution, the median (interquartile, IQR) was present. Numbers of patients in different subgroups were described as values and ratios. The Spearman rank correlation was utilized to identify correlations between time to treatment and immunological treatment response types, with Spearman r > 0.60 considered as strong correlations. All analyses were implemented by using R software, version 4.2.2 (R Foundation for Statistical Computing).

## 3 Results

### 3.1 Demographic and Clinical Characteristics

This study included 37 patients from 16 provinces and 31 cities around China, comprising 14 males (37.8%) and 23 females (62.8%), including 2 minors, with a male‐to‐female ratio of approximately 1: 2. Epilepsy was the most common phenotype, affecting 15 patients (40.5%), followed by limbic encephalitis (7 cases, 18.9%), overlap syndromes (7 cases, 18.9%), peripheral neuropathy (3 cases, 8.1%), brainstem dysfunction (2 cases, 5.4%), parkinsonism (2 cases, 5.4%), and cerebellar ataxia (1 case, 2.7%). Notably, Epilepsy and limbic encephalitis were predominant among the phenotypes observed in this study, whereas cerebellar ataxia and stiff person syndrome were more common in patients with overlap syndromes. There were isolated cases of brainstem dysfunction, parkinsonism, or peripheral neuropathy, highlighting distinct phenotype distributions compared to previous studies. Median age of symptom onset for all patients was 41.5 (25–59) years, with a median interval of 9 (1.5–36) months from onset to definitive diagnosis. Table 1 summarizes these patient characteristics.

**TABLE 1:** Characteristics and responses to immunotherapy of 37 patients with GAD65 neurological autoimmunity.

	Epilepsy (*n* = 15)	Limbic encephalitis (*n* = 7)	Cerebellar ataxia (*n* = 1)	Brainstem dysfunction (*n* = 2)	Parkinsonism (*n* = 2)	Peripheral neuropathy (*n* = 3)	Overlap syndromes (*n* = 7)	Total
Female/Male, *n*	11/ 4	5/ 2	1/ 0	2/ 0	0/ 2	0/ 3	4/ 3	23/ 14
Median ages of symptom onset (IQR)	24 (18.5–38.5)	44 (34–56)	61	48.5	64.5	68 (62–70)	41.5 (35–58)	41.5 (25–59)
Median time from symptom onset to definitive diagnosis in months (IQR)	14 (2.5–60)	2 (1–3.5)	9	5	66	1 (1–3.5)	20 (14.5–31)	9 (1.5–36)
Median time from symptom onset to first immunotherapy in months (IQR)	15 (2.8–60)	1.5 (1–2.75)	9	5.5	66	1.25	20 (10–31)	8.5 (1.5–37.5)
Systemic autoimmunity (%)								
Hashimoto's thyroiditis (%)	4/ 15 (26.7)	0/ 7 (0.0)	0/ 1 (0.0)	2/ 2 (100.0)	0/ 2 (0.0)	1/ 3 (33.3)	4/ 7 (57.1)	11/ 37 (29.7)
T1DM (%)	2/ 15 (13.3)	0/ 7 (0.0)	0/ 1 (0.0)	0/ 2 (0.0)	0/ 2 (0.0)	0/ 3 (0.0)	1/ 7 (14.3)	3/ 37 (8.1)
Median serum anti‐GAD65 titer (IQR)	1:320 (1:100–1:320)	1:320 (1:100–1:320)	1:320	1:480	1:210	1:64 (1:56–1:73)	1:320 (1:210–1:640)	1:320 (1:100–1:320)
Median CSF anti‐GAD65 titer (IQR)	1:320 (1:100–1:320)	1:320 (1:155–1:320)	1:640	1:640	1:210	1:64 (1:48–1:64)	1:320 (1:210–1:640)	1:320 (1:100–1:640)
CSF‐specific OCB (%)	7/ 15 (46.7)	5/ 7 (71.4)	1/ 1 (100.0)	2/ 2 (100.0)	2/ 2 (100.0)	0/ 3 (0.0)	6/ 7 (85.7)	23/ 37 (62.2)
Elevated 24‐h intrathecal IgG synthesis rates (%)	2/ 15 (13.3)	1/ 7 (14.3)	0/ 1 (0.0)	0/ 2 (0.0)	0/ 2 (0.0)	2/ 3 (66.7)	3/ 7 (42.9)	8/ 37 (21.6)
Loss to follow‐up (%)	0/ 15 (0.0)	2/ 7 (28.6)	0/ 1 (0.0)	0/ 2 (0.0)	0/ 2 (0.0)	1/ 3 (33.3)	1/ 7 (14.3)	4/ 37 (10.8)
Death	0/ 15 (0.0)	0/ 5 (0.0)	0/ 1 (0.0)	0/ 2 (0.0)	0/ 2 (0.0)	1/ 2 (33.3)	0/ 6 (0.0)	1/ 32 (3.1)
Follow‐up in months (IQR)	20 (16.5–26.5)	21 (18.5–37)	39	37	14	9.8	28 (14.5–55)	20.5 (16–37.3)
Response to immunotherapy								
Good response	4/ 15 (8.9)	3/ 5 (42.9)	0/ 1 (0.0)	0/ 2 (0.0)	0/ 2 (0.0)	1/ 1 (100.0)	0/ 6 (0.0)	8/ 32 (25.0)
Partial response	9/ 15 (60.0)	1/ 5 (14.3)	0/ 1 (0.0)	1/ 2 (50.0)	1/ 2 (50.0)	0/ 1 (0.0)	6/ 6 (100.0)	18/ 32 (56.3)
Mild response	1/ 15 (6.7)	0/ 5 (0.0)	0/ 1 (0.0)	0/ 2 (0.0)	1/ 2 (50.0)	0/ 1 (0.0)	0/ 6 (0.0)	2/ 32 (6.3)
No response	1/ 15 (6.7)	1/ 5 (14.3)	1/ 1 (100.0)	1/ 2 (50.0)	0/ 2 (0.0)	0/ 1 (0.0)	0/ 6 (0.0)	4/ 32 (12.5)
Epileptogenic focus resection (%)	1/ 15 (6.7)	1/ 5 (14.3)	0/ 1 (0.0)	0/ 2 (0.0)	0/ 2 (0.0)	0/ 1 (0.0)	0/ 6 (0.0)	2/ 32 (6.3)

Abbreviations: CSF, cerebrospinal fluid; OCB, oligoclonal bands; T1DM, Type1 diabetes mellitus.

### 3.2 Laboratory Characteristics

Five patients showed elevated white cell counts in CSF, with a median of 15 (13–33) × 10^6^/L (reference range: 0–8 × 10^6^/L) and mild protein elevation was observed in 5 patients with a median of 53 (49–54) mg/dL (reference range: 15–45 mg/dL). CSF‐specific OCB were present in 23 patients (62.2%). Eight patients exhibited elevated 24‐h intrathecal IgG synthesis rates with a median of 16.9 (11.2–23.9) mg/24 h (reference range: 0–9 mg/24 h).

Using the CBA method, all 37 patients tested positive for serum anti‐GAD65 antibodies with a median titer of 1:320 (1:100–1:320). In CSF, the positivity rate was 91.9%, with a median titer of 1:320 (1:100–1:640). Among them, 6 patients had serum and CSF anti‐GAD65 antibody concentrations exceeding 1000 U/mL (reference value: < 5 U/L). Immunoblotting assays confirmed positive results in both serum and CSF for 8 patients. Five patients also tested positive for other neuronal antibodies, including anti‐GABABR CV2, SOX1, Hu, Ri, Titin, and amphiphysin antibodies, with 3 cases showing combined positivity for anti‐SOX1 antibodies and 3 patients displaying dual or more antibody positivity. Nine patients underwent re‐evaluation for anti‐GAD65 antibodies at a median interval of 10 (6–19) months after immunotherapy; only 1 patient showed a negative conversion of serum antibody, while the remaining 8 patients retained positivity in serum (median 1:100, 1:100–1:320) and CSF (median 1:100, 1:100–1:320) with stable levels.

Systemic antibody testing revealed positive anti‐thyroid peroxidase (TPO) antibodies/anti‐thyroglobulin antibodies of 16 patients (43.2%), with both antibodies positive in 10 cases. Based on thyroid function tests, ultrasound findings, and endocrinology consultations, 11 patients (29.7%) were diagnosed with Hashimoto's thyroiditis, including 1 patient diagnosed with Sjögren's syndrome due to longstanding xerostomia, xerophthalmia, and positive anti‐nuclear antibodies, anti‐SSA antibodies, and anti‐Ro‐52 antibodies. Seven patients (18.9%) tested positive for serum anti‐nuclear antibodies, and 3 patients were positive for anti‐Ro‐52 antibodies. Three patients (8.1%) were diagnosed with T1DM and received regular subcutaneous insulin therapy.

### 3.3 Diverse Clinical Presentations of GAD65 Neurological Autoimmunity

In our cohort, epilepsy patients were primarily young and middle‐aged, with diverse seizure types. There were significant differences among patients in the median time from onset to definitive diagnosis and initiation of immunotherapy, as well as in their responses to immunotherapy. Some patients experienced significant improvement with steroids or IVIg alone, while a few patients who received multiple treatments only achieved slight improvement. One 62‐year‐old female patient with limbic encephalitis was found to have anti‐GAD65, CV2, and GABABR antibodies in both serum and CSF. This patient was transferred to the Neuro Intensive Care Unit (NICU) due to the severity of symptoms. PET/CT indicated a possible lung tumor, and paraneoplastic limbic encephalitis could not be ruled out. Unfortunately, the patient was lost to follow‐up.

In this cohort, two female patients presented exclusively with brainstem dysfunction. One patient experienced dizziness, diplopia, right‐sided ptosis, intermittent nausea, and vomiting. Despite initial treatment with intravenous methylprednisolone (IVMP), IVIg, and MMF, which was discontinued due to inadequate symptom relief and intolerable side effects, she eventually became bedridden and lost independence (see Table 2. Patient 1). The other patient started with vertigo and developed persistent diplopia, with physical examination revealing left eye abduction and limited downward gaze. Treatment included IVMP and sequential rituximab (RTX), leading to gradual symptom improvement (see Table 2. Patient 2).

**TABLE 2:** Detailed description on patients with brainstem dysfunction and parkinsonism.

	Gender, age (years old)	Serum antibodies	CSF antibodies	Clinical manifestation	CSF characteristics	Imaging findings	Time interval from symptom onset to initiating immunotherapy (months)	Immunotherapy	Follow‐up duration and outcomes
Patient 1	Female, 66	GAD65‐IgG 1: 640	GAD65‐IgG 1: 640	Brainstem dysfunction	Pleocytosis: 10 × 10^6^/L (reference range: 0–8 × 10^6^/L); protein: 49 mg/dL (reference range: 15–45 mg/dL); OCB patterns: type II	MR imaging of brain and spinal cord were normal	10	IVIg 0.4 g/kg/day for 5 days, IVMP 1 g/day for 5 days, then oral prednisone followed by slow tapering Oral MMF	Followed up for a total of 36 months, with symptoms deterioration gradually
Patient 2	Female, 32	GAD65‐IgG 1: 320	GAD65‐IgG 1:640	Brainstem dysfunction	Oligoclonal band patterns: type II	MR imaging of brain and spinal cord were normal	1	IVMP 1 g/day for 5 days, then oral prednisone followed by slow tapering RTX for three times	Followed up for a total of 38 months, with near‐complete recovery
Patient 3	Male, 63	GAD65‐IgG 1:100	GAD65‐IgG 1:100	Parkinsonism	OCB patterns: type II	MRI findings were consistent with the imaging characteristics of PSP (see Figure 1C), MR imaging of spinal cord was normal	48	IVMP 1 g/day for 5 days, then oral prednisone followed by slow tapering	Followed up for a total of 11 months, with partial recovery
Patient 4	Male, 77	GAD65‐IgG 1:100	GAD65‐IgG 1:100	Parkinsonism	OCB patterns: type II	Brain MR imaging was abnormal (see Figure 1D)	84	Initial IVIg 0.4 g/kg/day for 5 days, followed by intermittent IVIg Unable to tolerate the side effects of steroids and MMF	Followed up for a total of 17 months, with mild recovery

Abbreviation: PSP, progressive supranuclear palsy.

**FIGURE 1:**
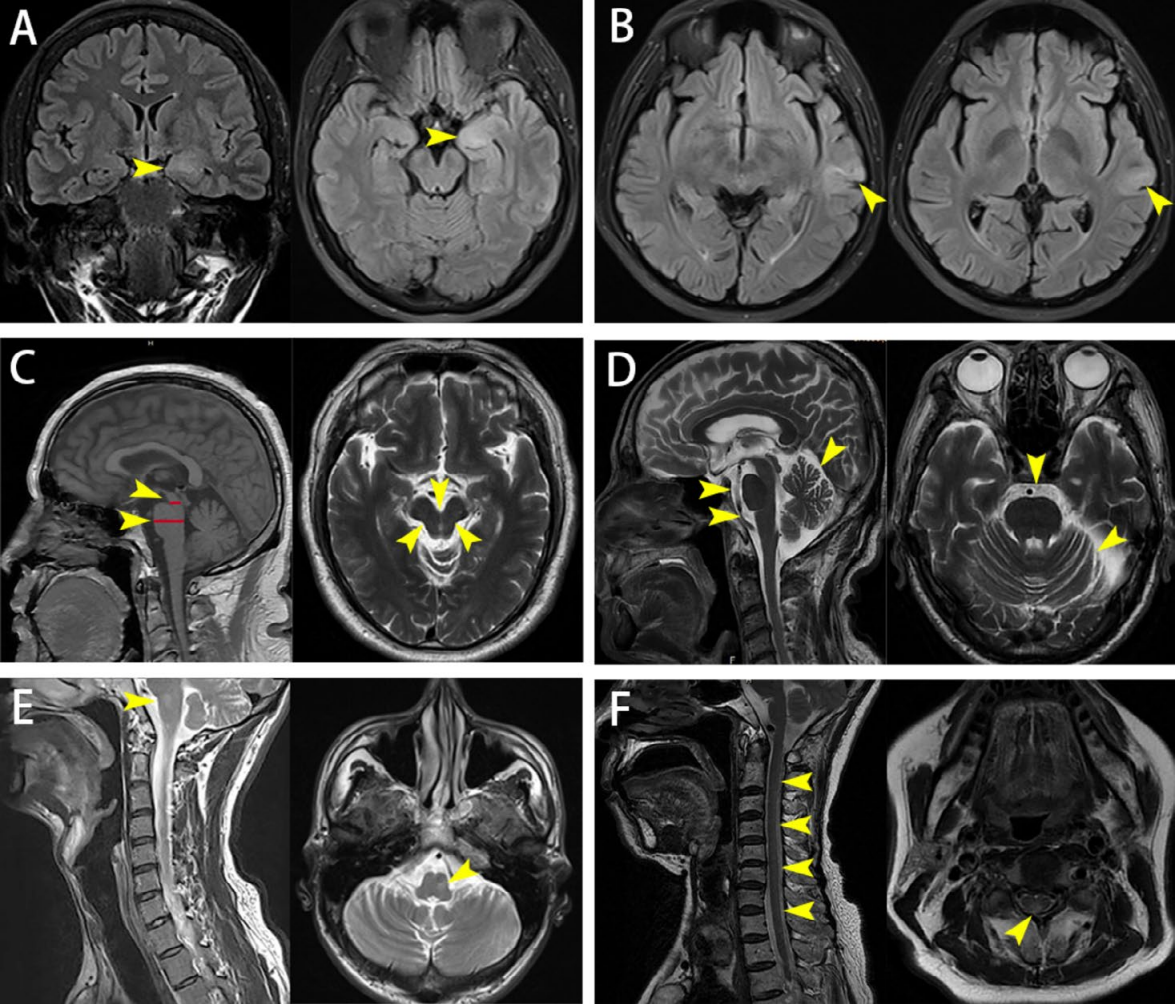
The distinctive MR imaging features of GAD65 neurological autoimmunity. (A) Fluid‐attenuated inversion recovery (FLAIR) imaging revealed swelling and hyperintensity in the left hippocampus and parahippocampal gyrus of a 33‐year‐old female patient with epilepsy. (B) Flair imaging demonstrated cortical thickening and hyperintensity in the left temporal lobe of a 24‐year‐old male patient with epilepsy. (C) T1‐weighted imaging (T1WI) and T2WI imaging revealed midbrain atrophy with the hummingbird sign in a 63‐year‐old male patient with a parkinsonian, showing the anteroposterior diameter ratio 0.41 of midbrain to pons. (D) T2WI imaging revealed widening of the pre‐pontine cistern and bilateral cerebellar subarachnoid spaces, along with mild atrophy of the left cerebellum, in a 77‐year‐old male patient with parkinsonian. (E) T2WI imaging revealed patchy abnormal signals in the left medulla of a 31‐year‐old male patient with overlap syndromes. (F) Cervical MRI showed dorsal strip‐like hyperintensity from C2 to T1 vertebral levels in a 69‐year‐old female with peripheral neuropathy.

Two male patients in this cohort displayed symptoms of parkinsonism. A 63‐year‐old male presented with bradykinesia, personality changes over 2 years, dysphagia, and declining memory over 6 months. Brain MRI indicated midbrain atrophy and the “hummingbird sign”, consistent with imaging features of progressive supranuclear palsy (PSP) (see Figure 1C). Treatment involved IVMP for 5 days followed by tapering oral steroid (OS) and concurrent MMF, resulting in significant symptom relief and restored independence (see Table 2. Patient 3). Another patient, a 77‐year‐old male, experienced 7 years of left limb bradykinesia and clumsiness unresponsive to levodopa. Initial treatment with IVIg provided slight improvement, but subsequent treatment with steroids and MMF was discontinued due to intolerable side effects (hallucinations, increased unsteady gait, and elevated transaminases). Symptomatic stability was maintained with intermittent IVIg without further deterioration (see Table 2. Patient 4 and Figure 1D). Notably, this patient repeated serum GAD65 antibody testing six times over 17 months, consistently showing titers at 1:100.

Four patients in this cohort suffered from peripheral nerve damage: one with subacute sensory neuropathy, one with subacute sensory neuropathy and myelopathy (see Figure 1F), one with Lambert‐Eaton myasthenic syndrome (LEMS), and one with peripheral neuropathy combined with HIV infection. PET‐CT or SPECT scans in the first three patients suggested the possibility of malignant lesions, accompanied by one or more additional neuronal antibodies. Two were lost to follow‐up, one did not receive immunotherapy and died approximately 3 months after discharge, and one patient with HIV infection experienced symptom disappearance after IVIg, supported by effective HIV treatment at a specialist hospital (see Table 3). It should be noted that the titers of GAD65 antibody in serum or CSF of these patients are relatively low compared to patients with other clinical phenotypes, with median titers of 1:64 (1:56–1:73) and 1:64 (1:48–1:64). Unfortunately, pathology data were unavailable for these patients.

**TABLE 3:** Detailed description on patients with peripheral neuropathy.

	Gender, age (years old)	Serum antibodies	CSF antibodies	Clinical manifestation	CSF characteristics	Imaging findings	Electrophysiology study	Time interval from symptom onset to initiating immunotherapy (months)	Follow‐up duration and outcomes
Patient 1	Female, 69	GAD65‐IgG 1:64 Hu‐IgG 1:320 Ri‐IgG 1:100 SOX1‐IgG 1:100 Titin‐IgG 1:64	GAD65‐IgG 1:64 Hu‐IgG 1:320 Ri‐IgG 1:64 SOX1‐IgG 1:32	Overlap syndromes: subacute sensory neuronopathy, paraneoplastic Myelopathy	Pleocytosis: 33 × 10^6^/L (reference range: 0–8 × 10^6^/L); 24 h intrathecal IgG synthesis rate: 59.29 mg (reference range: 0–9 mg/24 h) OCB patterns: type II	Cervical MR imaging was abnormal (see Figure 1F), MR imaging brain and thoracic were normal PET/CT was abnormal (see Figure 2F)	NCS indicated involvement of peripheral sensory fibers in all limbs	18	Loss to follow‐up
Patient 2	Male, 56	GAD65‐IgG 1:100 SOX1‐IgG 1:100	SOX1‐IgG 1:32	PNS: LEMS	Pleocytosis: 8 × 10^6^/L; protein: 54 mg/dL (reference range: 15–45 mg/dL)	SPECT was abnormal (see Figure 2G)	NCS indicated significant reduction of CMAP amplitude in all limbs. RNS indicates a decrement in amplitude of the right ulnar nerve at low frequency (3 Hz) and an increment reaching 1161% at high frequency (50 Hz, 500 times)	1	Loss to follow‐up
Patient 3	Male, 68 HIV infection	GAD65‐IgG 1:64	GAD65‐IgG 1:64	Peripheral neuropathy, primarily involving sensory nerves	Pleocytosis: 13 × 10^6^/L; IgG: 19.1 mg/dL (reference range: 0.48–5.86 mg/dL); 24 h intrathecal IgG synthesis rate: 35.63 mg/24 h	MR imaging of brain and spinal cord were normal	NCS indicated involvement of peripheral sensory fibers in all limbs	1.5	Followed up for a total of 16 months, with complete improvement
Patient 4	Male, 72	GAD65‐IgG 1:32 Amphiphysin‐IgG 1:320 Hu‐IgG 1:320 GABA_B_R‐IgG 1:100	GAD65‐IgG 1:32 Amphiphysin‐IgG 1:320 Hu‐IgG 1:100 GABA_B_R‐IgG 1:320	PNS: Subacute sensory neuronopathy	Pleocytosis: 15 × 10^6^/L; protein: 78 mg/dL; 24 h intrathecal IgG synthesis rate: 15.70 mg/24 h	PET/CT reveals abnormal hypermetabolism in the gastric antrum, pylorus and a nodule of the inferior lobe of right lung, with a strong suspicion of malignancy	NCS indicated involvement of peripheral sensory fibers in all limbs, with a marked predominance in both lower limbs	Not receiving immunotherapy	Died at 3 months of follow‐up

Abbreviations: CMAP, compound muscle action potential; HIV, human immunodeficiency virus; IgG, Immunoglobulin G; LEMS, Lambert‐Eaton myasthenic syndrome; NCS, nerve conduction studies; PNS, paraneoplastic neurological syndromes; RNS, repetitive nerve stimulation; SPECT, single photon emission computed tomography.

Seven patients were diagnosed with overlap syndromes, comprising 18.9% of the cohort and encompassing six overlap patterns. These included combinations such as cerebellar ataxia with limbic encephalitis, stiff person syndrome, and brainstem dysfunction, peripheral neuropathy with myelopathy, as well as epilepsy with stiff person syndrome or brainstem dysfunction.

### 3.4 Imaging Findings

All patients underwent comprehensive brain MRI evaluations, revealing abnormalities in 21 cases, none of which were gadolinium‐enhanced. The hippocampus and/or hippocampal gyrus (71.43%) and temporal lobes (33.33%) were predominantly affected, with involvement also noted in the brainstem, cerebellum, amygdala, insula, basal ganglia, and corona radiata, predominantly showing high signal intensity on T2‐weighted imaging. Hippocampal atrophy was most common, some accompanied by brainstem or cerebellar atrophy, with isolated cases exhibiting hippocampal swelling and thickening of the temporal lobe cortex (see Figure 1A). One patient with overlap syndromes of cerebellar ataxia, stiff person syndrome, and brainstem dysfunction had brainstem T2‐hyperintensity indicative of brainstem involvement (see Figure 1E).

**FIGURE 2:**
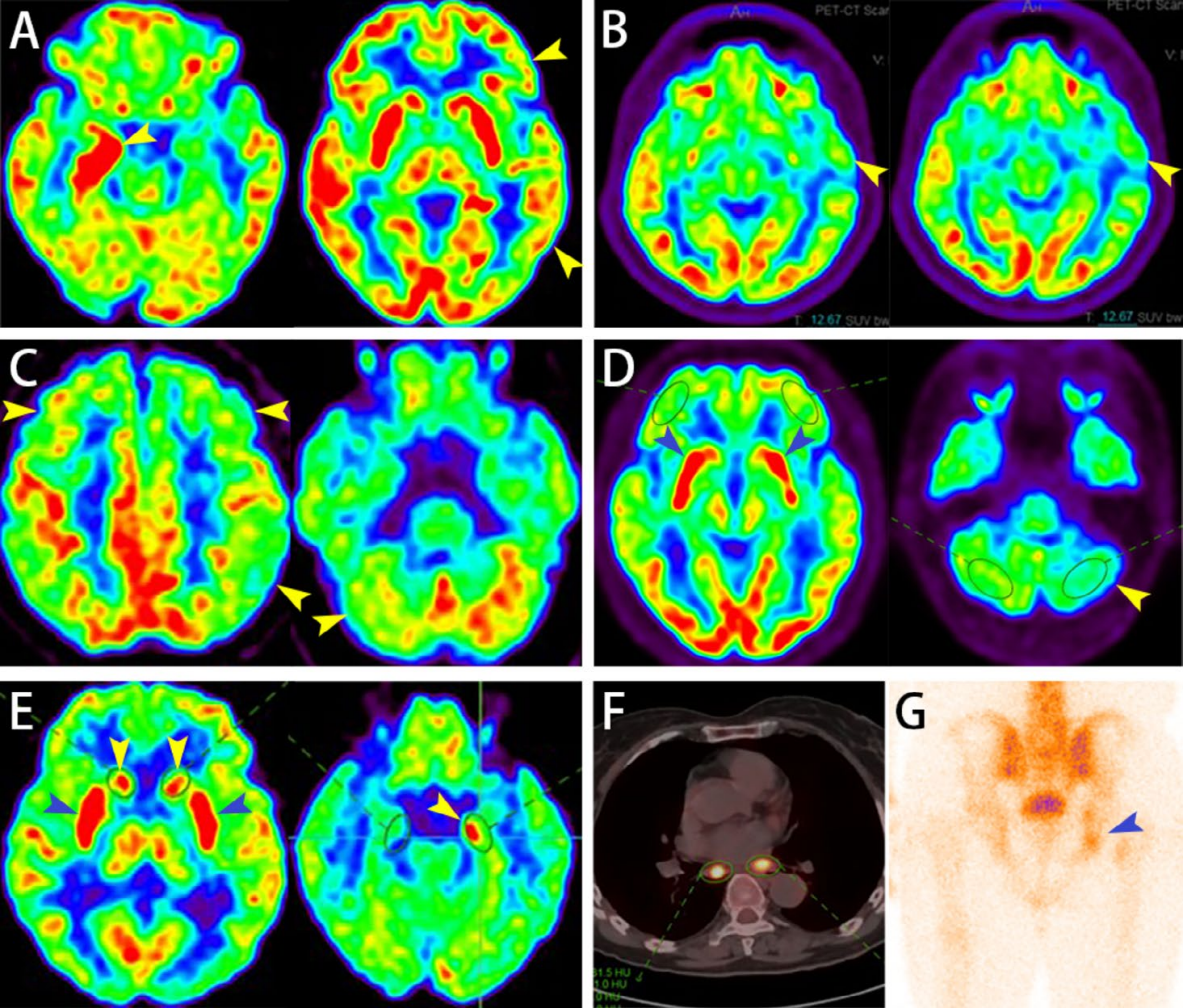
The distinctive PET‐CT/SPECT imaging features of GAD65 neurological autoimmunity. (A) A 50‐year‐old female patient with limbic encephalitis exhibits abnormal hypermetabolism in the right hippocampus and hypometabolism in the left frontal and temporal lobes. (B) A 24‐year‐old male patient with epilepsy exhibits prominent hypometabolism in the right temporal lobe cortex, corresponding with the lesion region on the brain MRI (see Figure 1B). (C) A 63‐year‐old male patient with parkinsonism demonstrates mild to severe hypometabolism in the bilateral frontal, parietal, and temporal lobes, most notably in the left frontal, parietal, and parieto‐occipital junction, with mild hypometabolism in the right cerebellar hemisphere (His brain MR imaging; see Figure 1C). (D) A 77‐year‐old male patient with parkinsonism demonstrates hypermetabolism in the bilateral caudate nuclei and moderate hypometabolism in the left cerebellum hemisphere (His brain MR imaging see Figure 1D). (E) A 62‐year‐old female patient with limbic encephalitis exhibits abnormal hypermetabolism in the bilateral basal ganglia and the left hippocampal. (F) A 69‐year‐old female patient with peripheral neuropathy revealed hypermetabolism of the multiple enlarged lymph nodes in both pulmonary hila and mediastinum. (G) SPECT revealed abnormally hypermetabolism in the right acetabulum and localized ischium of a 56‐year‐old female patient with LEMS, with tumor metastasis not excluded. LEMS, Lambert‐Eaton Myasthenic Syndrome.

Twelve patients underwent PET/CT scans, revealing metabolic abnormalities in various brain regions for 10 patients: temporal lobe (80%), frontal lobe (70%), parietal lobe (70%), basal ganglia (40%), occipital lobe (30%), hippocampus (30%), and cerebellum (20%). Among these, 7 patients showed hypometabolism in ≥ 1 brain region, including 2 cases of epilepsy, 2 cases of overlap syndromes, 2 cases of limbic encephalitis, and 1 case of parkinsonism (e.g., Figure 2B,C), while 3 patients exhibited both hypo‐ and hypermetabolic regions, including 2 cases of limbic encephalitis (see Figure 2A,E) and 1 case of parkinsonism (see Figure 2D). Among the 4 patients with peripheral neuropathy, two with subacute sensory neuropathy showed hypermetabolism regions in the lungs (see Figure 2F), with one also showing hypermetabolism lesions in the gastrointestinal tract, suggesting potential malignant lesions. One patient diagnosed with Lambert‐Eaton myasthenic syndrome showed abnormal bone metabolic activity in the right acetabulum and right ischium on single photon emission computed tomography (SPECT), with metastasis not ruled out (see Figure 2G).

### 3.5 Treatment and Prognosis

Thirty‐six patients (97.3%) received immunotherapy within a median time of 8.5 (1.5–37.5) months from symptom onset (see Table 1). Four cases were lost to follow‐up, leaving a median follow‐up period of 20.5 (16–37.3) months among the remaining 32 patients (see Table 4). Acute‐phase treatments involved IVMP, IVIg, and PLEX, with 15 patients (46.9%) receiving IVMP alone, 5 patients (15.6%) receiving IVIg alone, 12 patients (37.5%) receiving combination or sequential treatments of IVMP, IVIg, or PLEX, but only one patient underwent all three treatments sequentially. Maintenance‐phase treatments involved MMF or RTX, with 9 patients (28.1%) receiving MMF alone or with OS, 5 patients (15.6%) receiving RTX infusion (periodic single dose of 375 mg/m2 of body surface area; reinfusion will be given when CD19+ B‐cell counts of total lymphocytes reach 1%) alone or with OS, and 5 patients (15.6%) initially receiving MMF without marked improvement, converting to RTX infusion, then 3 patients with epilepsy experiencing a reduction in seizure frequency and less severity in seizure types, and one patient with limbic encephalitis achieving complete remission.

**TABLE 4:** Detailed description of immunotherapy responses of 32 patients with GAD65 neurological autoimmunity who received immunotherapy and completed follow‐up.

	Good response	Partial response	Mild response	No response	Total
Patients number (*n*) (%)	8 (25.0)	18 (56.3)	2 (6.3)	4 (12.5)	32 (100.0)
Gender (*n*) (%)					
Male	3 (37.5)	6 (33.3)	1 (50.0)	1 (25.0)	11 (34.4)
Female	5 (62.5)	12 (66.7)	1 (50.0)	3 (75.0)	21 (65.6)
Median time from symptom onset to definitive diagnosis in months (IQR)	1.25 (1.00–7.50)	23.00 (4.00–46.50)	49.00 (31.50–66.50)	9.00 (7.50–27.75)	
Median time from symptom onset to first immunotherapy in months (IQR)	1.50 (1.38–4.13)	23.00 (2.50–46.50)	49.50 (32.25–66.75)	9.50 (7.50–28.50)	
Acute phase treatment					
IVMP only (%)	3 (37.5)	10 (55.6)	1 (50.0)	1 (25.0)	15 (46.9)
IVIg only (%)	2 (25.0)	2 (11.1)	0 (0.0)	1 (25.0)	5 (15.6)
IVMP and IVIg (%)	3 (37.5)	5 (27.8)	1 (50.0)	2 (50.0)	11 (34.4)
IVMP and IVIG and PLEX (%)	0 (0.0)	1 (5.6)	0 (0.0)	0 (0.0)	1 (3.1)
Maintenance phase treatment					
MMF alone or with OS (%)	1 (12.5)	5 (27.8)	1 (50.0)	2 (50.0)	9 (28.1)
RTX alone or with OS (%)	1 (12.5)	3 (16.7)	1 (50.0)	0 (0.0)	5 (15.6)
MMF converted to RTX (%)	1 (12.5)	3 (16.7)	0 (0.0)	1 (25.0)	5 (15.6)
Relapse during the follow‐up (%)	0 (0.0)	11 (61.1)	2 (100.0)	4 (100.0)	17 (53.1)

Abbreviations: IVIg, intravenous immunoglobulin; IVMP, intravenous methylprednisolone; MMF, mycophenolate mofetil; OS, oral steroid; PLEX, plasma exchange; RTX, rituximab.

**FIGURE 3:**
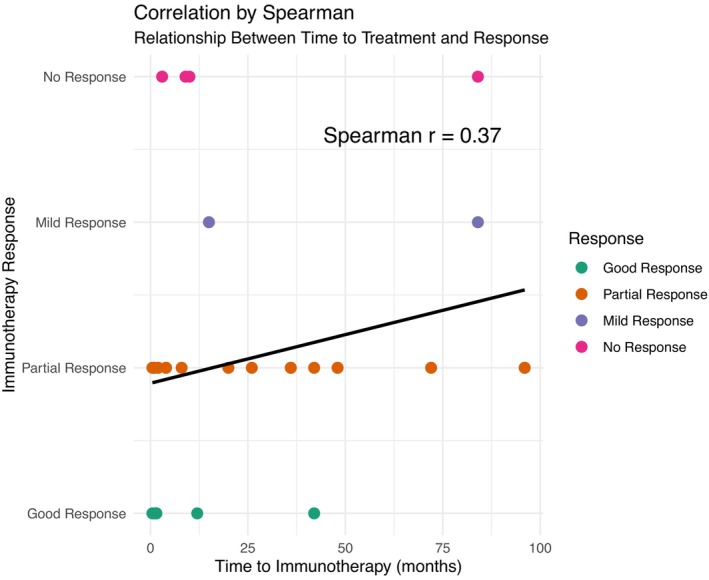
Analysis of the correlation between clinical prognosis and the timing of immunotherapy initiation.

Most patients responded favorably to immunotherapy, including good response (*n* = 8) and partial response (*n* = 18). Notably, among the 8 patients with good response, including 4 with epilepsy and 3 with limbic encephalitis accompanied by seizures, all received concurrent antiepileptic drug (see Tables 1 and 4). However, 2 patients with epilepsy and parkinsonism, respectively showed only mild response, 4 patients with limbic encephalitis accompanied by seizures, cerebellar ataxia, brainstem dysfunction, and epilepsy respectively, did not respond to immunotherapy, experiencing ongoing symptom deterioration (see Table 1). One patient with epilepsy and another with limbic encephalitis in the aforementioned 6 patients underwent various treatments, including IVMP, MMF, and RTX, without marked improvement, and were seizure‐free following epileptogenic focus resection. Spearman's rank correlation was used to assess the relationship between median time from symptom onset to first immunotherapy and response types, but only weak correlation was found, as shown in Figure 3.

## 4 Discussion

In our cohort study, female patients predominated, showcasing a diverse clinical spectrum that includes exceptionally rare or previously unreported phenotypes: brainstem dysfunction, parkinsonism, and peripheral neuropathy. The median age of onset was younger among epilepsy patients, whereas patients with peripheral neuropathy and parkinsonism exhibited older onset ages. No significant age difference was observed in limbic encephalitis or overlap syndromes. A high proportion of patients tested positive for both serum and CSF GAD65‐IgG antibodies, distinguishing it from conditions such as neuromyelitis optica spectrum disorders, or anti‐NMDAR receptor encephalitis, where antibodies are typically found predominantly in either serum or CSF [13, 14]. Among the nine patients retested after several months, 8 cases maintained positive GAD65‐IgG titers, indicating persistent antibody presence over an extended period with minimal likelihood of seroconversion. This contrasts with the conclusions of a previous study, which reported a decrease in antibody titers following immunotherapy [15]. Nearly three‐quarters of the patients exhibited a type 2 oligoclonal band pattern, with approximately one‐quarter concurrently diagnosed with Hashimoto's thyroiditis, suggesting potential systemic immune abnormalities. Notably, only a minority of patients showed mildly elevated levels of CSF white cells and protein, indicating intrathecal GAD65Ab production, rather than a compromised blood–brain barrier (BBB) [16].

Previous studies often regard brainstem dysfunction as a secondary manifestation associated with other clinical phenotypes [5]. In our cohort, however, two patients manifested solely with brainstem dysfunction without typical concurrent phenotypes, marking their first report. While brainstem dysfunction combined with other clinical phenotypes may aid diagnosis, isolated brainstem dysfunction complicates it. This suggests that even in cases presenting solely with brainstem dysfunction, GAD65 neurological autoimmunity should be considered, thereby refining antibody testing.

Parkinsonism, represents a rare clinical phenotype observed exclusively in serum GAD65 antibody‐positive subjects [6]. Patients in our study with parkinsonism exhibited onset ages and clinical features consistent with Parkinson's disease (PD), posing significant diagnostic challenges, one of them potentially characterized by long‐term unilateral involvement. A 60‐year‐old male patient presented with left hemiparkinsonism, with positive GAD65 antibodies in serum and CSF, and his symptoms remained stable with regular IVIg [7]. This indicates that parkinsonism/hemiparkinsonism may represent an early manifestation of prototype syndromes or a novel phenotype, possibly involving the GABAergic dysfunction of basal ganglia or the upper brainstem, suggesting a potential link between regional GABA + levels and pathology progression in PD patients [17, 18, 19]. Such cases prompt swift investigation into GAD65 neurological autoimmunity, especially when patients develop subacute or chronic ataxia and show poor response to levodopa. In some instances, GAD65 neurological autoimmunity should be considered in differential diagnoses for parkinsonism or even atypical parkinsonism alongside conditions like multiple system atrophy, corticobasal degeneration, or PSP. Prompt recognition is paramount because early immunotherapy may confer a better prognosis.

Previous studies primarily implicate GAD65 antibodies in affecting GABAergic neurons in the CNS, with unclear effects on the peripheral nervous system. Previously, only one case of cranial nerve and peripheral nerve damage in an underage patient and two cases of Miller Fisher syndrome in adults were reported with positive serum GAD65 antibodies [20], which were detected by ELISA, and their titers were relatively low. In our cohort, however, four adult patients presented with peripheral nerve involvement, three of whom had multiple anti‐neuronal antibodies, and one of whom had spinal cord involvement and corresponding symptoms. These findings and an absence of GAD expression in the peripheral nervous system suggest that low‐titer GAD‐ab detected in our patient and others may not be pathogenic or markers for a neurological disorder but rather the reflection and bystander effect of the ongoing immune reaction against the peripheral nerves. Such presumably nonpathogenic low GAD‐ab levels have also been detected in other autoimmune neurological disorders such as myasthenia gravis and multiple sclerosis and have been considered merely as nonspecific markers of an inflammatory condition [4].

Previous research suggests that patients with a phenotype of cerebellar ataxia typically exhibit progressive cerebellar atrophy on brain MRI [4]. Among the 4 patients in our cohort presenting with features of cerebellar ataxia, MRI in only one case indicated slight deepening of the right cerebellar fissure compared to the contralateral side. During the acute/subacute phase in epilepsy or limbic encephalitis, the most common abnormalities included hyperintensity on T2/FLAIR sequences and unilateral or bilateral “swelling” of the medial temporal structures [21, 22, 23, 24], resembling MRI findings in herpes simplex virus (HSV) encephalitis, which may regress over time or progress to persistent hyperintensity on follow‐up imaging, eventually transitioning to hippocampal atrophy or sclerosis [25]. In our study cohort, most patients with epilepsy or limbic encephalitis exhibited abnormal signals in the hippocampus and/or temporal lobes, with or without volume reduction; isolated cases indicated hippocampal swelling or thickening of the temporal lobe cortex. This variability in imaging findings may be attributed to the disease course [26], as not all patients in our cohort underwent standardized or uniform imaging examinations during the initial stages of illness. Overall, PET/CT primarily showed hypometabolism in cortical regions including parietal, frontal, occipital lobes, temporal, and hippocampus, which was consistent with previous reports [27, 28, 29]. Only 2 cases with the acute phase of limbic encephalitis and 1 case with parkinsonism exhibited hypermetabolism in the hippocampus, basal ganglia, and putamen. There was huge diversity in metabolic patterns among different phenotypes. Moreover, the timing of imaging, seizures, and acute treatments can alter the PET patterns strongly.

There was significant heterogeneity in the immunotherapy response among patients with GAD65 neurological autoimmunity. Patients with epilepsy positive for GAD65 antibodies may experience varying degrees of improvement with immunotherapy; however, in the long term, most patients continued to have seizures even after lesionectomy [5]. Notably, two epilepsy patients in our cohort remained seizure‐free post‐surgery, suggesting variability in lesion localization accuracy and surgical skill levels. Interestingly, some epilepsy patients benefited significantly from late‐initiated immunotherapy with steroids or IVIg alone, whereas early‐initiated, multi‐drug immunotherapy sometimes yielded limited clinical improvement, suggesting a need for further exploration into underlying mechanisms. There was a significant disparity in the time from symptom onset to initiation of immunotherapy among patients with different clinical phenotypes. Peripheral neuropathy and limbic encephalitis patients tended to initiate immunotherapy more promptly, compared to those with overlap syndromes, epilepsy, and parkinsonism, possibly due to certain clinical presentations prompting physician awareness or limitations in recognition of certain diseases.

## 5 Conclusion

The spectrum of GAD65‐related autoimmune neurological diseases appeared to be broader than previously understood, encompassing a range of clinical presentations with varying responses to immunotherapy. Patients can benefit from immunotherapy regardless of when it is started; however, early immunotherapy should be strongly encouraged. RTX may be more effective than MMF, however, this requires more rigorous prospective studies to explore. For rare phenotypes such as PD‐like presentations, if clinical manifestations or treatment responses diverge from the initial diagnosis, it is crucial to consider alternative possibilities and conduct thorough investigations to avoid diagnostic delays. Peripheral neuropathy often coexists with other neuronal antibodies, yet the precise pathogenic mechanisms and the role of GAD65 antibodies remain unclear and require further investigation.

## Conflicts of Interest

The authors declare no conflicts of interest.

## Data Availability

The data that support the findings of this study are available from the corresponding author upon reasonable request.
